# Association between peripapillary OCTA parameters and non-proliferative diabetic retinopathy: a cross-sectional study

**DOI:** 10.1186/s40942-026-00914-2

**Published:** 2026-07-31

**Authors:** Ru Zhou, Jun Zhang, Zichen Wang, Yiyi Chen, Wei Dong, Yanhan Fan, Bin Yang

**Affiliations:** Department of Ophthalmology, People’s Hospital of Yangzhong City, No. 235 Yangzi Middle Road, Yangzhong City, Zhenjiang, Jiangsu 212200 China

**Keywords:** Diabetic retinopathy, Optical coherence tomography angiography, Peripapillary, Nerve fiber layer vascular plexus vessel density (NFLVPVD)

## Abstract

**Purpose:**

This study aimed to compare peripapillary OCTA parameters between eyes with no diabetic retinopathy (NDR) and those with non-proliferative diabetic retinopathy (NPDR), and to investigate their independent associations with NPDR status.

**Methods:**

This cross-sectional study enrolled 128 healthy controls and 116 patients with type 2 diabetes mellitus. Diabetic eyes were categorized into the NDR group (*n* = 41 eyes) and mild, moderate, and severe NPDR groups (*n* = 32, 23, and 20 eyes, respectively). All subjects underwent peripapillary OCTA scanning. We quantitatively analyzed nerve fiber layer vascular plexus vessel density (NFLVPVD) and superficial vascular plexus vessel density (SVPVD), as well as peripapillary retinal nerve fiber layer (pRNFL) thickness. Baseline characteristics and OCTA quantitative parameters were compared across the control, NDR, and three NPDR subgroups. Multivariable-adjusted logistic regression was then performed to examine the association between peripapillary OCTA parameters and NPDR.

**Results:**

Compared with the NDR group, NFLVPVD, SVPVD, inferior 3-mm pRNFL thickness, and superior 6-mm pRNFL thickness were significantly lower in the pooled NPDR group (all *P* < 0.05). In multivariable-adjusted binary logistic regression, higher NFLVPVD was independently associated with lower odds of NPDR (OR = 0.884, 95% CI: 0.800–0.977, *P* = 0.016).

**Conclusions:**

Peripapillary NFLVPVD is independently associated with lower odds of NPDR.

**Supplementary Information:**

The online version contains supplementary material available at https://doi.org/10.1186/s40942-026-00914-2.

## Introduction

China has the world’s largest diabetic population, which poses a substantial public health burden. Diabetes mellitus (DM), a chronic metabolic disease, exhibits a continuously rising prevalence [1]. Diabetic retinopathy (DR), one of the major complications of DM, is the leading cause of visual impairment among working-age adults [2] and has attracted extensive research attention in ophthalmology and endocrinology. Current evidence points to two core pathological processes driving DR: retinal microvascular damage and neuronal degeneration. These changes, driven by chronic hyperglycemia, are frequently accompanied by programmed cell death within the retina. These pathological alterations disrupt retinal structural homeostasis and may ultimately result in irreversible visual dysfunction [3]. For this reason, characterizing the interrelationship between retinal microvascular and neural structural parameters in relation to DR status is essential to understanding the underlying pathophysiology and to informing future clinical strategies.

As a non-invasive imaging modality, optical coherence tomography angiography (OCTA) enables efficient and reproducible visualization of fundus blood flow and has become widely used in the assessment and monitoring of diabetic retinal vascular lesions. Beyond three-dimensional visualization of retinal and choroidal vascular structures, OCTA also quantifies parameters such as vessel density, providing objective metrics that reflect diabetes-induced microvascular remodeling [4–7]. The peripapillary region is a key anatomical location for the evaluation of retinal neurovascular coupling. Its unique radial vascular structure of capillaries parallel to the retinal nerve fiber axons supplies metabolic perfusion to the peripapillary nerve fiber layer [8]. This specialized structural feature renders this region highly sensitive to diabetic microcirculatory dysfunction and early retinal neurovascular injury. Yet, the bulk of current research has centered on macular vascular analysis, leaving peripapillary microvascular and structural data in diabetic patients comparatively sparse and inconsistent. Although several studies have identified peripapillary microcirculatory changes in diabetic patients [8–12], reports on peripapillary retinal nerve fiber layer thickness in diabetes remain conflicting.

We designed this study to carry out a quantitative comparison of nerve fiber layer vascular plexus vessel density (NFLVPVD), superficial vascular plexus vessel density (SVPVD), and peripapillary retinal nerve fiber layer (pRNFL) thickness between type 2 diabetes mellitus (T2DM) patients with no diabetic retinopathy (NDR) and those presenting with non-proliferative diabetic retinopathy (NPDR). In parallel, we also set out to determine whether these parameters remained independently associated with NPDR after adjustment for a range of clinical confounders.

## Methods

### Study design

This study was a single-center, cross-sectional observational study, and all procedures were performed in accordance with the principles of the Declaration of Helsinki. The research protocol was approved by the Ethics Committee of Yangzhong People’s Hospital (Approval No. 2023130), and written informed consent was obtained from all participants prior to enrollment.

## Study population

### Study group

We enrolled patients with type 2 diabetes mellitus (T2DM) admitted to the Department of Endocrinology at the People’s Hospital of Yangzhong City from November 2024 to May 2025. All patients met the following inclusion criteria: (1) T2DM diagnosis confirmed by an endocrinologist; (2) aged 20 to 75 years; (3) retinal findings consistent with the grading criteria of the International Clinical Diabetic Retinopathy (ICDR) severity scale [13] for NDR or NPDR; (4) complete clinical records and high-quality OCTA images were available. All participants underwent fundus photography, supplemented by FFA when clinically indicated. After screening, a total of 116 patients (116 study eyes) were enrolled and stratified by retinopathy severity into four subgroups: NDR (41 eyes), mild NPDR (32 eyes), moderate NPDR (23 eyes), and severe NPDR (20 eyes). All patients fulfilled the universal exclusion criteria described below.

### Control group

Healthy volunteers undergoing lung and upper gastrointestinal cancer screenings at the People’s Hospital of Yangzhong City within the same enrollment period were recruited. After strict screening, 128 participants (128 study eyes) were assigned to the control group. The inclusion criteria were as follows: (1) able to complete questionnaires and full ophthalmic examinations, with high-quality OCTA images acquired; (2) aged 20 to 75 years; and (3) no self-reported history of DM or impaired glucose tolerance was recorded. All participants with glycated hemoglobin (HbA1c) reaching the diabetic threshold (≥ 6.5%) were excluded from the healthy control group.

### Universal exclusion criteria for all participants

(1) severe systemic diseases, including uncontrolled hypertension, severe cardiovascular or cerebrovascular diseases, stroke, malignant tumors, and renal dysfunction; (2) history of intraocular surgery, such as cataract surgery, glaucoma surgery, vitreoretinal surgery, retinal photocoagulation, or intravitreal anti-vascular endothelial growth factor (anti-VEGF) injections; (3) cognitive or psychiatric impairments precluding reliable ophthalmic examinations; (4) coexisting ocular diseases that may interfere with quantitative study metrics, including optic neuropathy, age-related macular degeneration, retinitis pigmentosa, and other retinal vascular disorders; (5) severe ocular media opacity that degrades fundus image quality; (6) high myopia with a spherical equivalent of ≤ − 6.00 diopters; (7) ungradable or suboptimal OCTA images unsuitable for quantitative analysis.

### Study Eye Selection Criteria

#### T2DM study group

The eye with more severe retinopathy was designated as the study eye. If the severity of retinopathy was identical in both eyes, one eye was selected randomly.

#### Healthy control group

One eye per participant was designated as the study eye. When refraction and fundus findings were identical between the two eyes, one eye was selected at random. In case there was a mild difference between the two eyes, the eye with completely normal ocular structures was chosen as the study eye.

### General data collection

Demographic and clinical information, including age, sex, medical history, and personal history such as diabetes duration, smoking status, and alcohol consumption, as well as anthropometric data (height and weight), were collected using a standardized health questionnaire. For anthropometric assessment, participants were measured while wearing light indoor clothing and slippers. Body mass index (BMI) was subsequently derived as weight in kilograms divided by the square of height in meters. Systolic and diastolic blood pressure readings were obtained after participants had rested in a seated position for a minimum of 15 min.

### Laboratory evaluation

On the day of eye examinations, all participants had venous blood sampling after fasting (≥ 8 h). HbA1c levels were measured in whole blood. The serum was separated by centrifugation and used for measurements of lipid profile, including low-density lipoprotein cholesterol (LDL-C), high-density lipoprotein cholesterol (HDL-C), total cholesterol (TC) and triglycerides (TG).

### Ophthalmic examination

All patients received best-corrected visual acuity (BCVA) testing. Visual acuity was converted from decimal to logarithm of the minimum angle of resolution (logMAR) for the statistical analysis with the formula log_10_ (1/VA). Other ophthalmological examinations included intraocular pressure, slit-lamp biomicroscopy, color vision, standardized 45° posterior pole fundus photography, and OCTA. The fundus photographs of all patients were centered on the macula with a 45° view of the central posterior retina. Fundus fluorescein angiography (FFA) was additionally conducted as an auxiliary grading tool whenever clinical judgment deemed it necessary.

A single experienced attending ophthalmologist graded the severity of diabetic retinopathy in strict accordance with the ICDR severity scale. To reduce measurement bias stemming from inconsistent operation, all OCTA image acquisitions were completed by one specially trained imaging technician.

### OCTA image acquisition and processing

#### Device and Scanning Parameters

All OCTA scans were acquired with a spectral-domain OCTA device (Spectralis, Heidelberg Engineering, Heidelberg, Germany). We adopted a 6 × 6 mm square scanning protocol centered on the optic disc; the device operates at an 870 nm wavelength with a scanning rate of 85,000 A-scans per second. Its axial resolution reaches 3.9 μm, and the transverse resolution is 6 μm.

## Quality control and subject exclusion

We only included OCTA scans with a quality score (Q score) of 25 or higher. The TruTrack eye-tracking module and the projection artifact suppression were activated during image acquisition to ensure accurate centration on the optic disc. When slight segmentation offsets were observed in individual scans, manual correction was applied before proceeding with quantitative measurements.

A total of 270 participants (270 eyes) underwent initial OCTA scanning. After applying the quality filters, 26 eyes from 26 participants were excluded—primarily due to insufficient quality (Q score < 25). Other reasons included extensive motion artifacts, media opacities, severe segmentation errors, or marked scan decentration. Ultimately, 244 participants (244 eyes) were included in the final analysis: 128 from healthy control subjects and 116 from patients with T2DM.

### Automatic image layer segmentation

After scanning, the OCTA system’s proprietary software automatically segmented images into the following vascular layers: the superficial vascular complex (SVC), comprising the nerve fiber layer vascular plexus (NFLVP) and superficial vascular plexus (SVP); the deep vascular complex (DVC), which is subdivided into the intermediate capillary plexus (ICP) and deep capillary plexus (DCP); the avascular complex layer (ACL); and the choroidal vascular layer (CVL). This study only analyzed superficial peripapillary plexuses closely linked to optic nerve perfusion (NFLVP and SVP); deep capillary complexes were not included in quantitative analysis.

### Quantitative measurement parameters

**Fig. 1:**
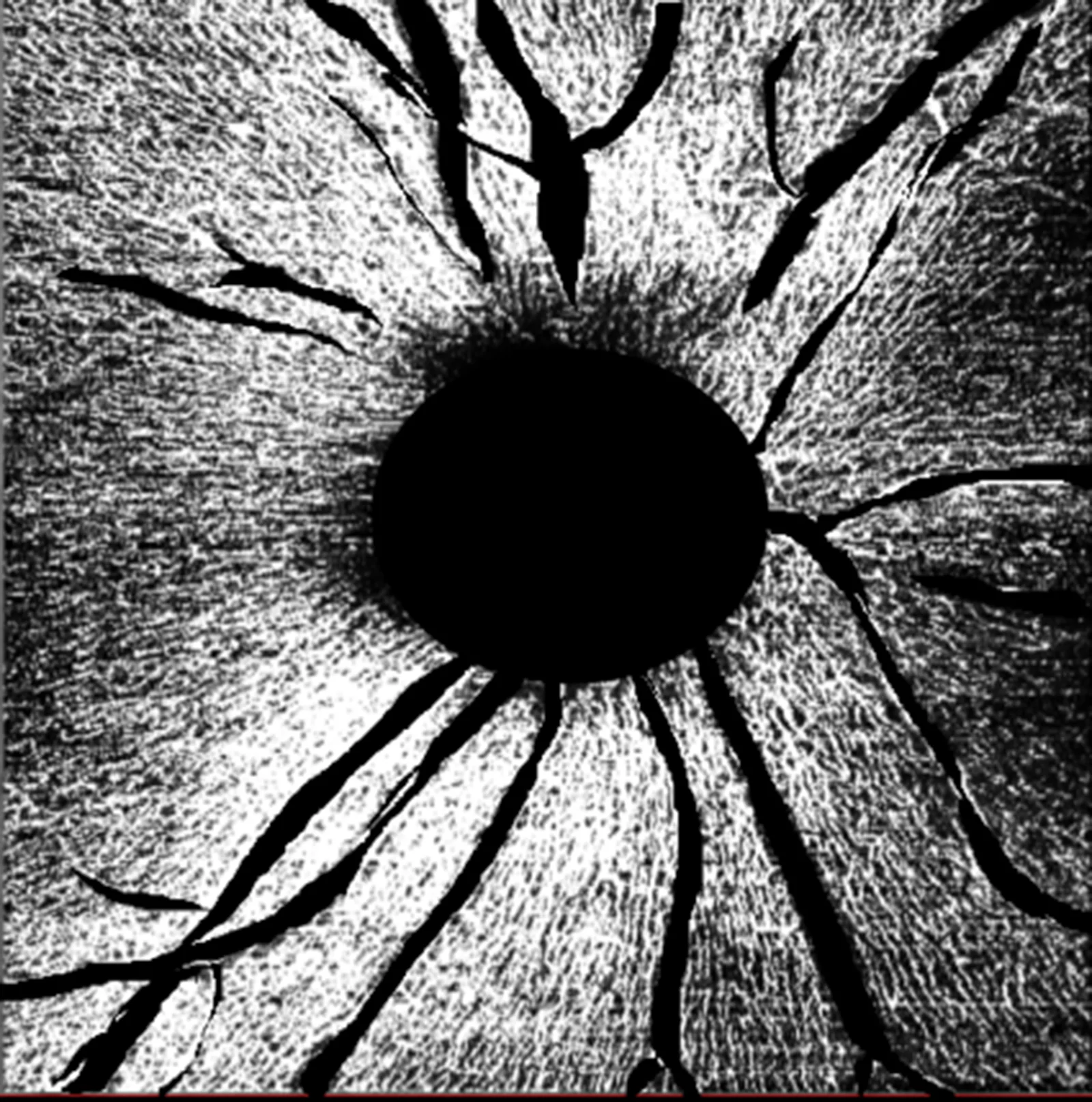
Peripapillary vascular density of the NFLVP after masking the optic disc and major retinal vessels

**Fig. 2:**
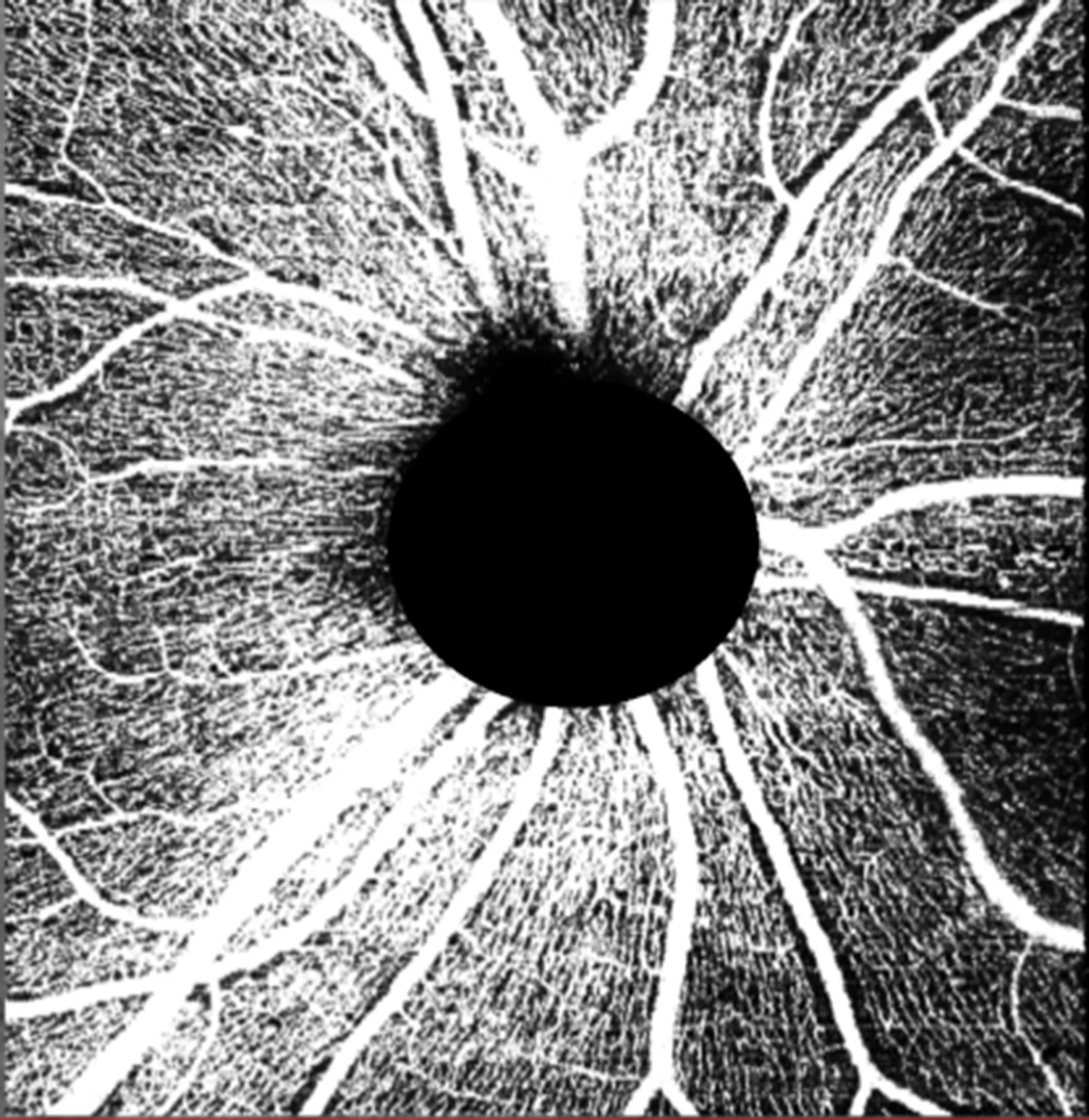
Global peripapillary vascular density of the SVP after masking the optic disc

**Fig. 3:**
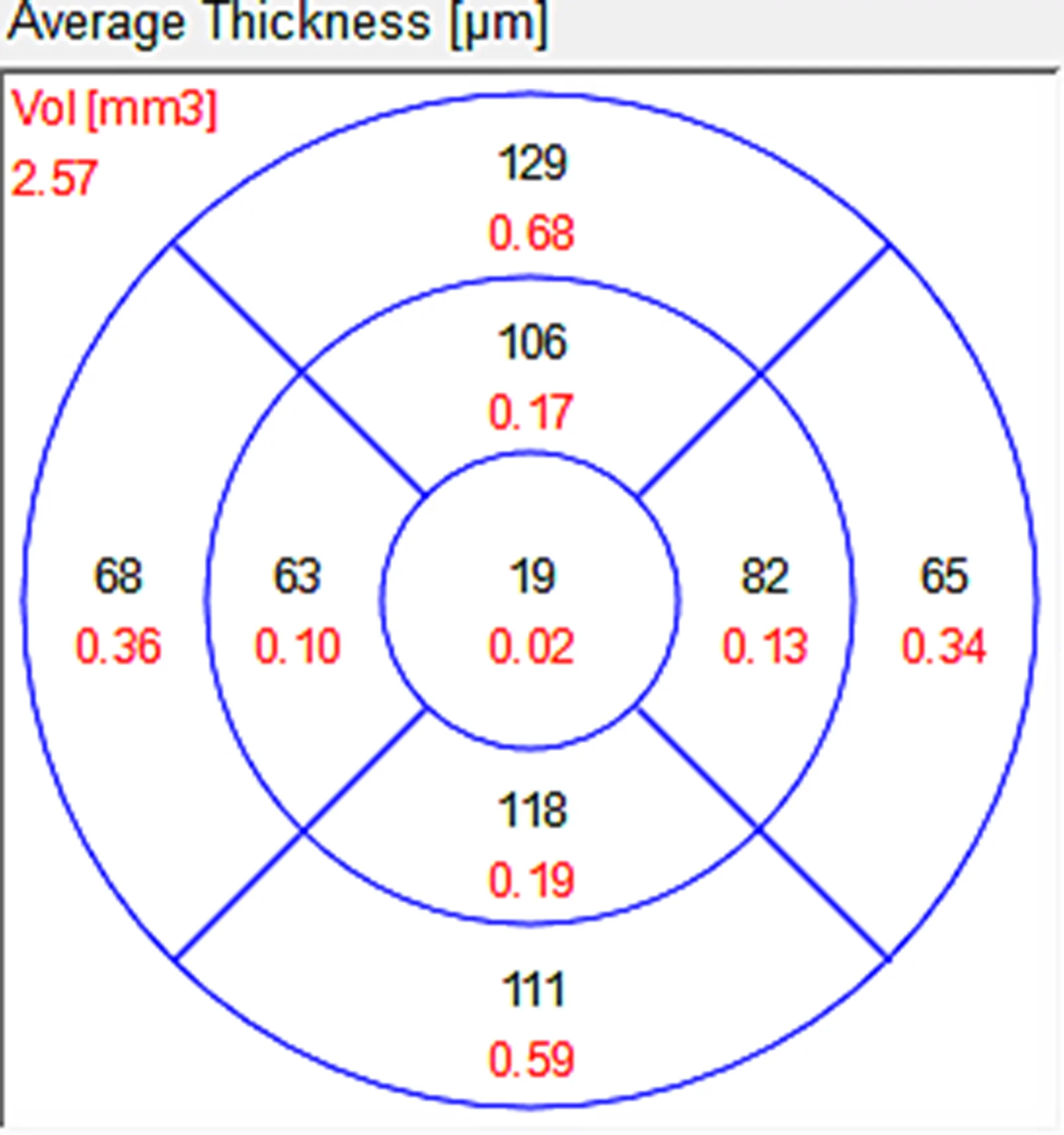
pRNFL thickness map: peripapillary annuli of 3 mm and 6 mm diameters, with measurements across four quadrants (superior, inferior, nasal, temporal)

The system automatically quantified pRNFL thickness from the structural optical coherence tomography volumetric scans across the superior, inferior, nasal, and temporal quadrants within 3-mm- and 6-mm- diameter annuli centered on the optic disc. Vessel density metrics of the NFLVP and SVP layers were subsequently quantified with ImageJ software (National Institutes of Health, Bethesda, MD, USA). The primary quantitative outcome indicators were as follows:

- 1. Peripapillary NFLVPVD, calculated after masking the optic disc and major retinal vessels (as detailed in the Image analysis section; Fig. 1).
- 2. Global peripapillary SVPVD, calculated after masking the optic disc (Fig. 2).
- 3. Quadrant-specific pRNFL thickness (superior, inferior, nasal, and temporal) (Fig. 3).

### Relevant definitions

Current smokers were defined as participants with a cumulative smoking history of ≥ 100 cigarettes who remained active smokers at enrollment, while non-smokers were those with a cumulative smoking exposure of < 100 cigarettes [14]. Regular alcohol consumption status was defined as alcohol intake at least once weekly [14].

### Standardized OCTA vascular layer definitions

According to the Spectralis system’s segmentation algorithm, the NFLVP captures blood flow signals from the internal limiting membrane to the retinal nerve fiber layer. The SVP algorithmically covers vascular signals extending from the retinal nerve fiber layer down to 17 μm beneath the inferior border of the inner plexiform layer. Vessel density was defined as the ratio of perfused pixels with detectable blood flow signals to the total pixels within the region of interest. This metric reflects the relative area occupied by perfused vessels within the peripapillary region.

### Image analysis

OCTA scans were loaded into ImageJ software and converted to a standard 8-bit grayscale image for quantitative measurement. To exclude interference from the optic disc and major retinal vessels during NFLVPVD quantification, these regions were manually delineated using the Rectangle Selection or Oval Selection tools and masked via the Edit > Clear function (pixel values reset to background). Major retinal vessels were defined as the primary (first-order) and secondary (second-order) trunks branching from the central retinal artery and central retinal vein visible on the OCTA en face slab. These thick primary and secondary vascular trunks were manually traced and excluded together with the optic disc, whereas fine tertiary and smaller capillary branches were retained for density calculation.

For SVPVD quantification, only the optic disc was masked, while major retinal vessels were retained. Figure 1 displays a representative processing output for the NFLVP, showing vessel density calculations after masking the optic disc and major retinal vessels. Peripapillary vessel density metrics (NFLVPVD and SVPVD) were then quantified on preprocessed images following widely accepted standard analytical protocols [15]. A trained masked grader (Ru Zhou) manually delineated the optic disc and surrounding major retinal vessels for all scans. The grader was fully masked to participants’ clinical data and grouping status. Thirty OCTA images were randomly sampled from the full dataset for reliability assessment. For intra-observer reliability, the same grader re-outlined and reanalyzed these images after a 2-week interval. For inter-observer reliability, a second independent grader quantified the identical set of 30 images. Intraclass correlation coefficients (ICCs) were calculated: the intra-observer ICC was 0.991, and the inter-observer ICC was 0.878. Both values reflected excellent measurement reliability.

### Statistical analysis

Statistical analyses were performed using SPSS 27.0 software (IBM Corp., Armonk, NY, USA). The Kolmogorov–Smirnov test was used to evaluate the normality of continuous variables. Normally distributed data were expressed as mean ± standard deviation. Between-group comparisons were conducted using an independent-samples t-test, and multiple-group comparisons were performed using one-way analysis of variance (ANOVA), with the least significant difference (LSD) test for post hoc pairwise comparisons. Non-normally distributed data were presented as median (interquartile range, IQR). The Kruskal–Wallis H test was adopted for overall comparisons across multiple groups, and pairwise intergroup differences were assessed with the Mann–Whitney U test after Bonferroni correction. Categorical variables were described as frequencies (percentages) and compared using the χ² test. Binary logistic regression was constructed to identify independent OCTA correlates of NPDR (reference group: NDR; NDR = 0, NPDR = 1). All models were fully adjusted for predefined confounders: age, sex, T2DM duration, HbA1c, logMAR visual acuity, BMI, TC, TG, HDL-C, LDL-C, smoking status, and alcohol consumption status. Stratified multivariable-adjusted models were sequentially built: single-metric adjusted models, dual vessel density combined models, dual structural pRNFL combined models, and a primary combined model incorporating the independent vascular metric and marginally significant structural metric. Model 7 was predefined as the primary fully adjusted combined model to derive the core conclusion of this study. A *P* < 0.05 was defined as statistically significant.

## Results

### Baseline characteristics

A total of 244 participants were included in the final analysis, including 116 patients with T2DM and 128 healthy control subjects. Patients with T2DM were further stratified into four subgroups according to the ICDR severity scale: no diabetic retinopathy (NDR, *n* = 41), mild non-proliferative diabetic retinopathy (mild NPDR, *n* = 32), moderate non-proliferative diabetic retinopathy (moderate NPDR, *n* = 23), and severe non-proliferative diabetic retinopathy (severe NPDR, *n* = 20) (Table 1).

Significant intergroup differences were found in age, HbA1c, duration of T2DM, logMAR visual acuity, TC, HDL-C, LDL-C, sex, and smoking status (all *P* < 0.05). Pairwise comparisons were conducted with two separate reference groups: the healthy control group and the NDR group. Superscript a indicates a significant difference relative to the control group, while superscript b represents a significant difference relative to the NDR group.

**Table 1:** Baseline clinical characteristics of all participants

Variables	Total (*n* = 244)	Controls (*n* = 128)	NDR (*n* = 41)	Mild NPDR (*n* = 32)	Moderate NPDR (*n* = 23)	Severe NPDR (*n* = 20)	H/χ²	*P*-value
Age (years)	60.00 (55.00, 65.75)	59.00 (55.00, 62.25)	59.00 (53.00, 66.00)	62.00 (53.75, 67.25)	66.00 (60.00, 67.00)^a, b^	63.00 (57.50, 70.25)^a, b^	12.080	0.017
HbA1c (%)	6.22 (5.49, 8.55)	5.54 (5.18, 5.89)	9.20 (7.19, 10.35)^a^	8.64 (7.57, 10.78)^a^	8.51 (6.90, 10.80)^a^	8.70 (7.23, 10.43)^a^	157.542	< 0.001
Duration of T2DM (years)	0.00 (0.00, 8.75)	0.00 (0.00, 0.00)	7.00 (2.00, 13.00)^a^	6.00 (1.25, 16.25)^a^	10.00 (2.00, 15.00)^a^	15.00 (10.00, 20.00)^a^	214.491	< 0.001
Visual acuity (logMAR)^*^	0.16 (0.00, 0.30)	0.10 (0.00, 0.22)	0.22 (0.00, 0.30)	0.10 (0.00, 0.28)	0.10 (0.10, 0.30)	0.26 (0.13, 0.37)^a, b^	11.702	0.020
BMI (kg/m^2^)	24.19 (22.04, 26.53)	24.51 (22.45, 26.77)	23.15 (21.42, 26.06)	24.18 (22.32, 27.16)	23.85 (20.81, 26.20)	24.52 (22.00, 26.33)	4.304	0.366
TC (mmol/L)	5.04 (4.31, 5.76)	5.28 (4.58, 5.78)	4.87 (4.19, 5.87)	5.10 (4.16, 5.74)	4.74 (3.78, 6.01)	4.06 (3.32, 4.43)^a, b^	20.105	< 0.001
TG (mmol/L)	1.54 (1.10, 2.24)	1.60 (1.15, 2.23)	1.41 (1.12, 2.19)	1.73 (1.19, 2.39)	1.40 (1.08, 1.95)	1.27 (0.84, 2.27)	2.622	0.623
HDL-C (mmol/L)	1.44 (1.21, 1.72)	1.52 (1.29, 1.82)	1.24 (1.15, 1.61)^a^	1.33 (1.18, 1.43)^a^	1.30 (1.11, 1.93)	1.32 (0.99, 1.64)^a^	21.065	< 0.001
LDL-C (mmol/L)	2.57 (2.04, 3.12)	2.68 (2.30, 3.12)	2.36 (2.04, 3.10)	2.66 (1.96, 3.45)	2.32 (1.91, 3.46)	1.84 (1.58, 2.22)^a, b^	17.983	0.001
Sex, n (%)							19.061	< 0.001
Male	115 (47.13)	44 (34.38)	23 (56.10)	22 (68.75)	13 (56.52)	13 (65.00)		
Female	129 (52.87)	84 (65.63)	18 (43.90)	10 (31.25)	10 (43.48)	7 (35.00)		
Smoking, n (%)							17.362	0.002
Non-smoker	193 (79.10)	110 (85.94)	25 (60.98)	21 (65.62)	21 (91.30)	16 (80.00)		
Current smoker	51 (20.90)	18 (14.06)	16 (39.02)	11 (34.38)	2 (8.70)	4 (20.00)		
Alcohol consumption status, n (%)							8.314	0.081
No regular drinking	203 (83.20)	113 (88.28)	32 (78.05)	27 (84.38)	18 (78.26)	13 (65.00)		
Regular drinking	41 (16.80)	15 (11.72)	9 (21.95)	5 (15.62)	5 (21.74)	7 (35.00)		

Non-normally distributed continuous data are expressed as median (interquartile range, IQR). Multi-group comparisons of continuous variables across the five subgroups were conducted via the Kruskal–Wallis test (statistic H), and pairwise post-hoc comparisons were conducted via the Mann–Whitney U test with Bonferroni correction

Categorical variables are presented as n (%), and intergroup comparisons were conducted via the Chi-square test (statistic χ²)

T2DM, type 2 diabetes mellitus; BMI, body mass index; HbA1c, glycated hemoglobin; logMAR, logarithm of the minimum angle of resolution; TC, total cholesterol; TG, triglyceride; HDL-C, high-density lipoprotein cholesterol; LDL-C, low-density lipoprotein cholesterol

^*^ Visual acuity was converted to logMAR units for statistical analysis

^a^ *P* < 0.05 versus the control group; ^b^ *P* < 0.05 versus the NDR group

### Peripapillary OCTA metrics

Significant overall intergroup differences across the five groups were detected for four peripapillary OCTA metrics (all *P* < 0.05), including NFLVPVD, SVPVD, inferior pRNFL thickness at 3-mm, and inferior pRNFL thickness at 6-mm. The results of post-hoc pairwise comparisons showed that compared with the NDR group, NFLVPVD and 3-mm inferior pRNFL thickness were significantly lower in mild, moderate, and severe NPDR subgroups, while SVPVD was significantly lower only in the severe NPDR subgroup; compared with healthy controls, NFLVPVD and 3-mm inferior pRNFL thickness were significantly lower in moderate and severe NPDR groups, and SVPVD, together with 6-mm inferior pRNFL thickness was significantly lower only in the severe NPDR group ( all *P* < 0.05 ) (Table 2).

**Table 2:** Comparison of peripapillary OCTA metrics across the five study groups

Variables	Total (*n* = 244)	Controls (*n* = 128)	NDR (*n* = 41)	Mild NPDR (*n* = 32)	Moderate NPDR (*n* = 23)	Severe NPDR (*n* = 20)	F/H	*P*-value
NFLVPVD (%)	30.05 ± 5.64	30.77 ± 5.19	31.82 ± 5.76	28.75 ± 4.83^b^	28.30 ± 6.21^a, b^	25.89 ± 6.30^a, b^	5.631	< 0.001
SVPVD (%)	26.34 ± 5.46	26.58 ± 5.29	27.86 ± 5.71	25.77 ± 6.01	25.48 ± 4.56	23.50 ± 5.18^a, b^	2.500	0.043
**pRNFL thickness within a 3-mm-diameter annulus (µm)**								
Superior	101.50 (82.00, 121.75)	105.00 (86.00, 123.00)	93.00 (81.00, 123.50)	102.50 (88.00, 121.00)	87.00 (76.00, 109.00)	90.50 (60.75, 125.75)	7.237	0.124
Inferior	107.63 ± 29.82	111.13 ± 28.70	115.90 ± 32.33	101.19 ± 27.42^b^	94.52 ± 27.12^a, b^	93.65 ± 29.66^a, b^	3.999	0.004
Nasal	59.50 (51.00, 74.75)	60.00 (50.25, 74.75)	61.00 (51.00, 78.50)	56.00 (53.25, 74.25)	61.00 (50.00, 70.00)	55.50 (41.25, 69.75)	2.664	0.615
Temporal	57.00 (50.00, 66.00)	57.00 (50.25, 65.00)	58.00 (52.50, 69.50)	57.00 (52.25, 63.75)	54.00 (50.00, 60.00)	56.00 (37.25, 66.75)	3.160	0.531
**pRNFL thickness within a 6-mm-diameter annulus (µm)**								
Superior	90.50 (79.00, 100.00)	91.00 (81.25, 100.00)	95.00 (87.00, 100.50)	86.50 (69.75, 95.25)	86.00 (76.00, 97.00)	87.50 (69.75, 100.75)	7.972	0.093
Inferior	99.50 (87.00, 109.00)	101.50 (93.00, 110.00)	94.00 (83.00, 111.00)	98.50 (83.50, 106.75)	98.00 (88.00, 102.00)	82.50 (70.25, 104.75)^a^	14.935	0.005
Nasal	45.00 (40.00, 52.00)	44.00 (40.00, 51.00)	45.00 (42.00, 59.50)	44.50 (38.50, 48.75)	48.00 (42.00, 53.00)	45.00 (38.50, 51.75)	6.603	0.158
Temporal	59.00 (54.25, 64.75)	60.00 (55.00, 64.00)	59.00 (51.00, 64.50)	59.00 (53.50, 61.00)	63.00 (55.00, 69.00)	57.00 (50.25, 64.75)	3.553	0.470

Normally distributed OCTA metrics are presented as mean ± standard deviation. Multi-group comparisons were conducted using one-way analysis of variance (ANOVA; F statistic), followed by post hoc pairwise comparisons using the least significant difference (LSD) test. Conversely, non-normally distributed metrics are expressed as median (interquartile range, IQR). Multi-group comparisons were conducted using the Kruskal–Wallis test (statistic H), with post hoc pairwise comparisons performed using the Mann–Whitney U test with Bonferroni correction

NFLVPVD, nerve fiber layer vascular plexus vessel density; SVPVD, superficial vascular plexus vessel density; pRNFL, peripapillary retinal nerve fiber layer thickness

^a^ *P* < 0.05 versus the control group; ^b^ *P* < 0.05 versus the NDR group

### Baseline characteristics of NDR and NPDR groups

The data shown in Table 3 indicate that, except for age (*P* = 0.04), the NDR and NPDR groups were comparable in all other baseline characteristics (all *P* > 0.05).

**Table 3:** Comparison of baseline clinical characteristics between the NDR and NPDR groups

Variables	Total (*n* = 116)	NDR (*n* = 41)	NPDR (*n* = 75)	U/χ²	*P*-value
Age (years)	62.00 (55.00, 67.00)	59.00 (52.50, 66.00)	62.00 (56.00, 68.00)	1182.500	0.040
HbA1c (%)	8.70 (7.26, 10.55)	9.20 (7.19, 10.35)	8.70 (7.30, 10.70)	1528.000	0.956
Duration of T2DM (years)	10.00 (2.00, 15.75)	7.00 (2.00, 13.00)	10.00 (3.00, 18.00)	1332.500	0.235
Visual acuity (logMAR)^*^	0.22 (0.10, 0.30)	0.22 (0.00, 0.30)	0.22 (0.10, 0.30)	1460.500	0.650
BMI (kg/m^2^)	23.86 (21.97, 26.35)	23.15 (21.42, 26.06)	24.22 (22.04, 26.45)	1319.500	0.207
TC (mmol/L)	4.73 (3.94, 5.61)	4.87 (4.19, 5.87)	4.67 (3.84, 5.47)	1349.000	0.276
TG (mmol/L)	1.44 (1.06, 2.30)	1.41 (1.12, 2.19)	1.51 (1.04, 2.31)	1501.000	0.833
HDL-C (mmol/L)	1.31 (1.14, 1.59)	1.24 (1.15, 1.61)	1.31 (1.14, 1.57)	1480.500	0.742
LDL-C (mmol/L)	2.33 (1.82, 3.11)	2.36 (2.04, 3.10)	2.25 (1.81, 3.34)	1422.500	0.507
Sex, n (%)					
Male	71.00 (61.21)	23.00 (56.10)	48.00 (64.00)	0.697	0.404
Female	45.00 (38.79)	18.00 (43.90)	27.00 (36.00)		
Smoking, n (%)					
Non-smoker	83.00 (71.55)	25.00 (60.98)	58.00 (77.33)	3.485	0.062
Current smoker	33.00 (28.45)	16.00 (39.02)	17.00 (22.67)		
Alcohol consumption, n (%)					
No regular drinking	90.00 (77.59)	32.00 (78.05)	58.00 (77.33)	0.008	0.930
Regular drinking	26.00 (22.41)	9.00 (21.95)	17.00 (22.67)		

Non-normally distributed continuous data are expressed as median (interquartile range, IQR). Two-group comparisons of continuous variables between the NDR and NPDR groups were conducted via the Mann–Whitney U test (statistic U)

Categorical variables are presented as n (%), and intergroup comparisons were conducted via the Chi-square test (statistic χ²)

T2DM, type 2 diabetes mellitus; BMI, body mass index; HbA1c, glycated hemoglobin; logMAR, logarithm of the minimum angle of resolution; TC, total cholesterol; TG, triglyceride; HDL-C, high-density lipoprotein cholesterol; LDL-C, low-density lipoprotein cholesterol

^*^ Visual acuity was converted to logMAR units for statistical analysis

#### Comparison of peripapillary OCTA metrics across control, NDR, and NPDR groups

Compared with the NDR group, the NPDR group showed significant reductions in NFLVPVD, SVPVD, inferior pRNFL thickness at the 3-mm annulus, and superior pRNFL thickness at the 6-mm annulus (all *P* < 0.05). In comparison with healthy controls, the NPDR group presented significantly lower NFLVPVD, together with thinner pRNFL in the 3-mm inferior, 6-mm inferior, and 6-mm superior annular regions (all *P* < 0.05) (Table 4).

**Table 4:** Pairwise comparisons of peripapillary OCTA metrics between NDR and NPDR groups, and between controls and NPDR group

Variables	Total (*n* = 116)	NDR (*n* = 41)	NPDR (*n* = 75)	t/U	*P*-value	Controls (*n* = 128)	NPDR (*n* = 75)	t/U	*P*-value
NFLVPVD (%)	29.25 ± 6.03	31.82 ± 5.76	27.85 ± 5.73	3.562	< 0.001	30.77 ± 5.19	27.85 ± 5.73	3.724	< 0.001
SVPVD (%)	26.06 ± 5.65	27.86 ± 5.71	25.08 ± 5.40	2.597	0.011	26.58 ± 5.29	25.08 ± 5.40	1.944	0.053
**pRNFL thickness within a 3-mm-diameter annulus (µm)**									
Superior	95.00 (79.25, 120.50)	93.00 (81.00, 123.50)	95.00 (78.00, 119.00)	1456.000	0.638	105.00 (86.00, 123.00)	95.00 (78.00, 119.00)	4018.000	0.053
Inferior	103.77 ± 30.69	115.90 ± 32.33	97.13 ± 27.79	3.280	0.001	111.13 ± 28.70	97.13 ± 27.79	3.392	< 0.001
Nasal	58.00 (51.00, 74.75)	61.00 (51.00, 78.50)	57.00 (51.00, 70.00)	1334.000	0.240	60.00 (50.25, 74.75)	57.00 (51.00, 70.00)	4437.500	0.369
Temporal	57.00 (50.00, 66.00)	58.00 (52.50, 69.50)	56.00 (50.00, 64.00)	1271.000	0.124	57.00 (50.25, 65.00)	56.00 (50.00, 64.00)	4411.000	0.335
**pRNFL thickness within a 6-mm-diameter annulus (µm)**									
Superior	89.50 (76.00, 98.00)	95.00 (87.00, 100.50)	86.00 (75.00, 97.00)	1120.000	0.016	91.00 (81.25, 100.00)	86.00 (75.00, 97.00)	3854.500	0.019
Inferior	97.00 (81.00, 106.00)	94.00 (83.00, 111.00)	97.00 (80.00, 106.00)	1379.000	0.360	101.50 (93.00, 110.00)	97.00 (80.00, 106.00)	3437.000	< 0.001
Nasal	45.00 (41.25, 53.75)	45.00 (42.00, 59.50)	45.00 (41.00, 52.00)	1273.000	0.126	44.00 (40.00, 51.00)	45.00 (41.00, 52.00)	4622.500	0.660
Temporal	59.00 (52.00, 65.00)	59.00 (51.00, 64.50)	59.00 (55.00, 65.00)	1455.500	0.636	60.00 (55.00, 64.00)	59.00 (55.00, 65.00)	4722.000	0.847

Normally distributed OCTA metrics are presented as mean ± standard deviation, and two-group comparisons were performed using the independent-samples t-test (t-statistic). Conversely, non-normally distributed OCTA metrics are expressed as median (interquartile range, IQR), and two-group comparisons were conducted via the Mann-Whitney U test (statistic U)

Two pairwise comparisons were performed: the first between the NDR and NPDR groups; the second between healthy control subjects and the NPDR group

NFLVPVD, nerve fiber layer vascular plexus vessel density; SVPVD, superficial vascular plexus vessel density; pRNFL, peripapillary retinal nerve fiber layer thickness

#### Logical analysis of peripapillary data in the NDR and NPDR groups

This study used binary logistic regression to explore the independent associations between peripapillary OCTA metrics and NPDR status. The binary outcome was defined as NPDR versus NDR. All models were fully adjusted for potential confounders, including age, HbA1c, T2DM duration, logMAR visual acuity, BMI, TC, TG, HDL-C, LDL-C, sex, smoking status, and alcohol consumption status.

Four OCTA parameters showing significant intergroup differences in the preceding comparative analysis (Table 4) were incorporated into multivariable-adjusted regression models: NFLVPVD, SVPVD, inferior pRNFL thickness in the 3-mm annular zone, and superior pRNFL thickness in the 6-mm annular zone.

Models 1 to 4 were constructed as single-metric multivariable-adjusted models. After full covariate adjustment, all four OCTA metrics were significantly associated with NPDR status. Specifically, higher peripapillary NFLVPVD (OR = 0.859, 95% CI: 0.781–0.945, *P* = 0.002), SVPVD (OR = 0.906, 95% CI: 0.833–0.987, *P* = 0.023), inferior 3-mm pRNFL thickness (OR = 0.975, 95% CI: 0.957–0.993, *P* = 0.007), and superior 6-mm pRNFL thickness (OR = 0.960, 95% CI: 0.930–0.991, *P* = 0.012) were each negatively associated with NPDR.

To avoid multicollinearity and separately evaluate vascular and structural OCTA parameters, stratified multivariable-adjusted models were further developed. Model 5 simultaneously incorporated the two vessel density metrics. After mutual adjustment and full covariate correction, only NFLVPVD remained independently associated with NPDR (OR = 0.870, 95% CI: 0.777–0.975, *P* = 0.017), while the association between SVPVD and NPDR became non-significant (*P* = 0.676).

Model 6 included the two structural pRNFL thickness metrics. Following mutual adjustment and full covariate correction, neither inferior 3-mm pRNFL thickness nor superior 6-mm pRNFL thickness showed a significant independent association with NPDR (all *P* > 0.05).

Model 7 was established as the primary combined model. Based on the above stratified analyses, the independently significant vascular metric (NFLVPVD) and the structural metric with borderline statistical significance (inferior 3-mm pRNFL thickness, *P* = 0.078) were included simultaneously with all predefined confounding covariates. The final model showed that NFLVPVD exhibited an independent negative association with NPDR (OR = 0.884, 95% CI: 0.800–0.977, *P* = 0.016), whereas inferior 3-mm pRNFL thickness only showed a marginal trend without statistical significance.

**Table 5:** Binary logistic regression analyses exploring associations between peripapillary OCTA metrics and NPDR status

Variables	Wald _χ²_	*P*-value	OR	95% CI (lower)	95% CI (upper)
**Model 1**					
NFLVPVD (%)	9.764	0.002	0.859	0.781	0.945
**Model 2**					
SVPVD (%)	5.150	0.023	0.906	0.833	0.987
**Model 3**					
Inferior pRNFL (3-mm zone, µm)	7.289	0.007	0.975	0.957	0.993
**Model 4**					
Superior pRNFL (6-mm zone, µm)	6.261	0.012	0.960	0.930	0.991
**Model 5**					
NFLVPVD (%)	5.701	0.017	0.870	0.777	0.975
SVPVD (%)	0.174	0.676	0.978	0.880	1.086
**Model 6**					
Inferior pRNFL (3-mm zone, µm)	3.114	0.078	0.982	0.962	1.002
Superior pRNFL (6-mm zone, µm)	1.797	0.180	0.976	0.942	1.011
**Model 7**					
NFLVPVD (%)	5.801	0.016	0.884	0.800	0.977
Inferior pRNFL (3-mm zone, µm)	3.104	0.078	0.983	0.964	1.002

Binary logistic regression was used to explore the associations between peripapillary OCTA metrics and NPDR. The binary outcome was defined as NPDR = 1 and NDR = 0

All models were adjusted for the following covariates: age, sex, T2DM duration, HbA1c, logMAR visual acuity, BMI, TC, TG, HDL-C, LDL-C, smoking status, and alcohol consumption status

Model 1–4: Separate multivariable-adjusted models, each containing one statistically significant OCTA metric identified from the NDR versus NPDR comparison in Table 4, with covariate adjustment

Model 5: Multivariable-adjusted model simultaneously incorporating two vessel density metrics (NFLVPVD and SVPVD) and all covariates

Model 6: Multivariable-adjusted model simultaneously incorporating two structural pRNFL thickness metrics (inferior pRNFL thickness in the 3-mm annular zone and superior pRNFL thickness in the 6-mm annular zone) and all covariates

Model 7: Primary combined multivariable-adjusted model incorporating NFLVPVD (the significantly associated vascular metric) and inferior pRNFL thickness in the 3-mm annular zone (the borderline-associated structural metric), with covariate adjustment, to explore their independent associations with NPDR

OR, odds ratio; 95% CI, 95% confidence interval

NFLVPVD, nerve fiber layer vascular plexus vessel density; SVPVD, superficial vascular plexus vessel density; pRNFL, peripapillary retinal nerve fiber layer thickness

## Discussion

The peripapillary region is a critical site for evaluating retinal neurovascular coupling owing to its unique radial anatomy [8]. Capillaries in this region run parallel to retinal nerve fiber axons and deliver essential nutrients to the pRNFL. Given that diabetes-induced microvascular and neuronal injury may disrupt this structure-function relationship [3, 16], we quantified NFLVPVD and SVPVD in the 6 × 6 mm optic disc-centered area, and pRNFL thickness within both 3-mm and 6-mm peripapillary annuli to characterize neurovascular alterations across patients with NDR and NPDR.

Quantitative assessment of peripapillary OCTA metrics yielded multiple key associations (Tables 2 and 4). First, four parameters exhibited significant intergroup differences across DR subgroups: NFLVPVD, SVPVD, inferior pRNFL thickness in the 3-mm peripapillary annulus, and inferior pRNFL thickness in the 6-mm peripapillary annulus (Table 2). After pairwise comparison between NDR and NPDR, 6-mm inferior pRNFL thickness lost statistical significance; the four indicators (NFLVPVD, SVPVD, 3-mm inferior pRNFL thickness, and 6-mm superior pRNFL thickness) were included in regression analysis. Specifically, NFLVPVD and 3-mm inferior pRNFL thickness were markedly lower across mild, moderate, and severe NPDR subgroups relative to patients with NDR; SVPVD was only significantly reduced in the severe NPDR subgroup compared with NDR (Table 2), while superior pRNFL thickness within the 6- mm peripapillary annulus was significantly thinner in the pooled NPDR group versus NDR (Table 4). Second, multivariable - adjusted logistic regression revealed distinct patterns of association between OCTA metrics and NPDR status (Table 5). Although the four above-mentioned OCTA parameters each displayed significant associations with NPDR in separate single-metric models, only NFLVPVD retained a stable independent association after mutual adjustment for the two vessel density indicators. When the two structural pRNFL thickness parameters were incorporated simultaneously, neither reached statistical significance, with inferior 3-mm pRNFL thickness showing only a marginal trend toward association (*P* = 0.078). The primary combined regression model further showed that lower NFLVPVD was independently associated with the presence of NPDR after adjusting for structural pRNFL parameters and all predefined clinical confounders. These findings collectively suggest that reduced peripapillary NFLVPVD was independently associated with NPDR after comprehensive adjustment for demographic, metabolic, and lifestyle confounding factors. This cross-sectional association reflects only the statistical relationship between peripapillary microvascular perfusion and NPDR; additional multicenter prospective studies are needed to validate this finding.

Prior OCTA studies investigating diabetic retinal neurovascular changes have predominantly focused on macular vascular plexuses and confirmed reduced capillary density in DR cohorts [4–7]. The present work extends the investigation to the peripapillary circulation and further stratifies retinal microvessels to quantify the NFLVP and SVP separately. Several earlier works have partially characterized peripapillary microcirculatory changes in patients with clinically evident DR yet carry notable methodological limitations. Liu et al. [8] reported altered 3-mm peripapillary vessel density when comparing patients with NDR and NPDR; however, their cohort lacked a healthy reference group and failed to adjust for lifestyle confounders, including smoking and alcohol consumption. Cao et al. [9] reported that patients with mild NPDR exhibited significantly lower mean peripapillary NFLVPVD compared with healthy controls and participants with NDR. Nevertheless, lipid profiles and personal lifestyle factors were not adjusted in their analysis. Our results are partially consistent with the findings of Cui et al. [10]. No significant difference in peripapillary vascular density between the control and NDR groups, while significant differences existed between NDR and NPDR groups (except in the temporal quadrant). Healthy control comparisons are presented only as a reference context in this work, with significant baseline imbalances in systemic metabolic markers between healthy and diabetic patients, and important conclusions are derived solely from the comparisons between NDR and NPDR subgroups.

Published reports regarding pRNFL thickness alterations in diabetic retinal lesions remain inconsistent. Some previous studies reported that pRNFL thickness does not change significantly in NPDR [11]. while our cross-sectional data identified two distinct structural abnormalities: significant inferior-quadrant pRNFL thinning within the 3-mm annulus across all mild, moderate, and severe NPDR subgroups relative to patients with NDR and reduced superior pRNFL thickness within the 6-mm annulus in the pooled NPDR cohort. Liu et al. [8] also documented inferior pRNFL thinning among patients with moderate and severe NPDR. Bhaskaran et al. [12] reported significant thinning of ARNFL and RNFL across all quadrants in patients with DR, demonstrating progressive RNFL attenuation alongside increasing DR severity. Notably, superior quadrant RNFL loss was identified as the earliest marker of incipient retinal neurodegeneration. The increased vulnerability of the inferior peripapillary zone may be attributed to relatively weaker blood perfusion at the inferior optic nerve head, rendering this region more susceptible to ischemic damage [17].

As the most superficial vascular network surrounding the optic disc, the NFLVP sustains metabolic supply to the pRNFL [8]. Our cross - sectional observations identified concurrent reductions in NFLVPVD and inferior 3-mm pRNFL thickness among patients with NPDR, and both metrics exhibited significant associations with NPDR status in independent single-metric, fully adjusted models; superior 6-mm pRNFL thickness also showed a significant crude association with NPDR status. Peripapillary microvascular vessel density and peripapillary retinal nerve fiber layer thickness may interact with each other during the progression of diabetic retinopathy. This pathological cascade may establish a bidirectional adverse cycle: microvascular hypoperfusion may be accompanied by neuronal impairment, whereas damaged neurons and glia cannot maintain a stable local microenvironment, thus further aggravating vascular dysfunction [18]. However, the cross-sectional design of the present study precludes a definitive causal inference and cannot elucidate the temporal sequence of vascular and neuronal changes. This mechanistic speculation cannot be validated by the current observational data alone. Of note, although SVPVD was significantly lower in NPDR than in NDR and showed a significant association in its separate single-metric adjusted model, this association disappeared after mutual adjustment alongside NFLVPVD. The observed associations only suggest that reduced NFLVPVD and thinner inferior 3-mm pRNFL are associated with NPDR status in this cohort.

This study has several methodological strengths. First, imaging was done with a standardized 6 × 6 mm peripapillary scan, which is wide enough for quantitative evaluation of peripapillary microvasculature and nerve fiber layers. Second, the NPDR cases were further categorized into mild, moderate and severe strata based on ICDR criteria, allowing stratified assessment of peripapillary neurovascular changes at different stages of DR. Third, we simultaneously assessed pRNFL thickness in both 3-mm and 6-mm peripapillary annuli, to fully capture structural nerve fiber damage across two scanning radii. Fourth, the analysis comprehensively combined systemic metabolic indicators, ocular structural parameters, and modifiable lifestyle covariates (smoking and alcohol consumption) into fully adjusted regression models, and stratified multivariable-adjusted models were constructed for separate assessment of independent effects of vessel density and structural nerve fiber metrics, offering a multidimensional perspective for probing cross-sectional correlates of NPDR.

Some limitations of this analysis are important to note. First, the cross-sectional study design is a limitation that does not allow for determination of causal relationships between OCTA metrics and NPDR status; causal relationships and temporal progression of lesions cannot be ascertained. Longitudinal prospective cohort studies are required to validate these correlative findings. Second, manual segmentation of the optic disc and large retinal vessels using ImageJ is operator-dependent, which may introduce minor measurement variability, even though strict intra- and inter-observer reliability testing was performed. Third, sample sizes for moderate and severe NPDR subgroups were relatively limited, and age - stratified subgroup analyses within each DR stage were not conducted. Fourth, the unbalanced sample size and notable baseline disparities (age, sex, smoking, metabolic profiles) between healthy controls and diabetic subjects may compromise the credibility of cross - group comparisons involving controls. All discrepant baseline factors were fully adjusted in regression models to reduce confounding bias, and our core conclusions solely rely on NDR vs. NPDR analyses, with healthy controls used only for auxiliary reference. Fifth, although all available clinical covariates were incorporated, residual confounding cannot be entirely excluded due to unmeasured variables including chronic ocular medication use, anti-diabetic treatment regimens, axial length, and ocular perfusion pressure. In addition, all participants were hospitalized patients; the relatively high median HbA1c of 9.20% in the NDR group indicated suboptimal glycemic control, which may potentially confound the observed associations. Sixth, the external generalizability of the associations observed is limited due to the single-center nature of this cohort and replication in large-scale multicenter studies is warranted. Seventh, in agreement with prior literature, no significant differences in peripapillary vessel density were observed between healthy controls and patients with NDR in the present study, and such discrepancies across reports may be partly due to unequal sample sizes between the groups and needs to be confirmed in larger, balanced cohorts. Eighth, an asymmetric eye-selection strategy was employed, choosing the eye with more severe retinal lesions for participants with T2DM and a randomly selected or structurally intact eye for healthy controls. This selection approach may artificially inflate the intergroup differences in peripapillary OCTA vessel density and pRNFL thickness, which may introduce potential selection bias.

## Conclusion

This cross-sectional study showed that NFLVPVD was independently associated with NPDR in the fully adjusted combined model and was the sole OCTA vascular parameter to remain.

Statistically significant. Significant intergroup disparities in NFLVPVD were observed between patients with NDR and those with NPDR, providing evidence of vessel differences across the diabetic retinal disease from NDR to NPDR. These findings should be validated in prospective, multicenter studies before any diagnostic or prognostic application can be considered.

Imaging modalities capable of automated segmentation of peripapillary capillaries for quantitative vessel density measurement should be prioritized in future studies.

## Supplementary Information

Below is the link to the electronic supplementary material.

Supplementary Material 1

Supplementary Material 2

Supplementary Material 3

## Data Availability

The datasets used and/or analyzed during the current study are available from the corresponding author upon reasonable request.

## Abbreviations

NDR: No diabetic retinopathy
NPDR: Non–Proliferative Diabetic Retinopathy
OCTA: Optical Coherence Tomography Angiography
DM: Diabetes Mellitus
HbA1c: Glycated hemoglobin
NFLVPVD: Nerve fiber layer vascular plexus vessel density
SVPVD: Superficial vascular plexus vessel density
pRNFL: peripapillary retinal nerve fiber layer
T2DM: Type 2 diabetes mellitus
DR: Diabetic Retinopathy
ICDR: International Clinical Diabetic Retinopathy
FFA: Fundus fluorescein angiography
anti‑VEGF: anti‑vascular endothelial growth factor
BMI: Body mass index
BCVA: Best‑corrected visual acuity
logMAR: logarithm of the minimum angle of resolution
SVC: Superficial vascular complex
NFLVP: Nerve fiber layer vascular plexus
SVP: Superficial vascular plexus
DVC: Deep vascular complex
ICP: Intermediate capillary plexus
DCP: Deep capillary plexus
ACL: Avascular complex layer
CVL: Choroidal vascular layer
ROI: Region of interest
ICC: Intraclass correlation coefficient
ANOVA: Analysis of variance
LSD: Least significant difference
IQR: Interquartile range
TC: Total cholesterol
TG: Triglyceride
HDL‑C: High‑density lipoprotein cholesterol
LDL‑C: Low‑density lipoprotein cholesterol
OR: Odds ratio
CI: Confidence interval
