# Supplementary material for: Association between peripapillary OCTA parameters and non-proliferative diabetic retinopathy: a cross-sectional study
Source: Int J Retina Vitreous. 2026 Jul 31;12:142. doi: 10.1186/s40942-026-00914-2 (PMC13715244; doi:10.1186/s40942-026-00914-2)
Supplement: Supplementary file 1 [file 40942_2026_914_MOESM1_ESM.pdf]

Supplementary Table 1: sensitivity analysis

Variable	*Wald* χ^2	*P* Value	*OR*	95%*CI*	
**NFLVPVD**	8.533	**0.003**	0.893	0.827	0.963
**Age (year)**	0.284	0.594	1.012	0.968	1.058
**Smoking (Yes)**	2.569	0.109	0.481	0.196	1.177
**SvpVD**	3.766	0.052	0.926	0.857	1.001
**Age (year)**	1.146	0.284	1.023	0.981	1.067
**Smoking (Yes)**	1.674	0.196	0.562	0.235	1.345
**Inferior peripapillary RNFL (3mm zone)(ul)**	6.583	**0.01**	0.98	0.966	0.995
**Age (year)**	0.589	0.443	1.017	0.974	1.061
**Smoking (Yes)**	2.017	0.156	0.529	0.22	1.274
**Superior peripapillary RNFL (6mm zone)(ul)**	4.294	**0.038**	0.973	0.947	0.998
**Age (year)**	1.186	0.276	1.023	0.982	1.066
**Smoking (Yes)**	3.507	0.061	0.429	0.177	1.04
**NFLVPVD**	5.224	**0.022**	0.899	0.821	0.985
**SvpVD**	0.085	0.771	0.986	0.898	1.083
**Age (year)**	0.269	0.604	1.012	0.968	1.057
**Smoking (Yes)**	2.326	0.127	0.492	0.198	1.224
**Inferior peripapillary RNFL (3mm zone)(ul)**	3.366	**0.067**	0.984	0.967	1.001
**Superior peripapillary RNFL (6mm zone)(ul)**	0.748	0.387	0.987	0.957	1.017
**Age (year)**	0.469	0.493	1.015	0.972	1.06
**Smoking (Yes)**	2.442	0.118	0.486	0.197	1.201
**NFLVPVD**	4.456	**0.035**	0.915	0.843	0.994
**Inferior peripapillary RNFL (3mm zone)(ul)**	2.448	0.118	0.987	0.971	1.003
**Age (year)**	0.049	0.825	1.005	0.96	1.052
**Smoking (Yes)**	2.365	0.124	0.49	0.197	1.216

Variables with *P* < 0.1 in the univariate analysis of the NDR and NPDR groups were included in the multivariate regression analysis

To enhance statistical adjustment for confounding factors, we included variables with P < 0.1 from the comparison of baseline characteristics between NDR and NPDR in Table 3 into a multivariate regression analysis for sensitivity analysis.Ultimately, smoking (p = 0.062) and age were simultaneously incorporated into the multivariate analysis.To further clarify the independent association between each indicator and NPDR, and to avoid collinearity interference between related indicators, subgroup analyses were performed: the vascular density-related indicators (NFLVPVD and

SVPVD) and the retinal nerve fiber thickness-related indicators were separately incorporated into the multivariate model along with age and smoking . The results showed that NFLVPVD (OR=0.899, 95%CI= 0.821- 0.985) was significantly associated with NPDR, while the inferior pRNFL thickness at 3 mm(OR=0.984 95%CI= 0.967-1.001) also maintained a significant association with NPDR.

Subsequently, representative indicators from the two types of parameters (vascular density and thickness) were selected to construct a combined multivariate model with age and smoking. The results consistently confirmed that NFLVPVD (OR=0.915, 95%CI= 0.843- 0.994) was an independent protective factor for NPDR.
