# Supplementary material for: Association between peripapillary OCTA parameters and non-proliferative diabetic retinopathy: a cross-sectional study
Source: Int J Retina Vitreous. 2026 Jul 31;12:142. doi: 10.1186/s40942-026-00914-2 (PMC13715244; doi:10.1186/s40942-026-00914-2)
Supplement: Supplementary file 2 [file 40942_2026_914_MOESM2_ESM.pdf]

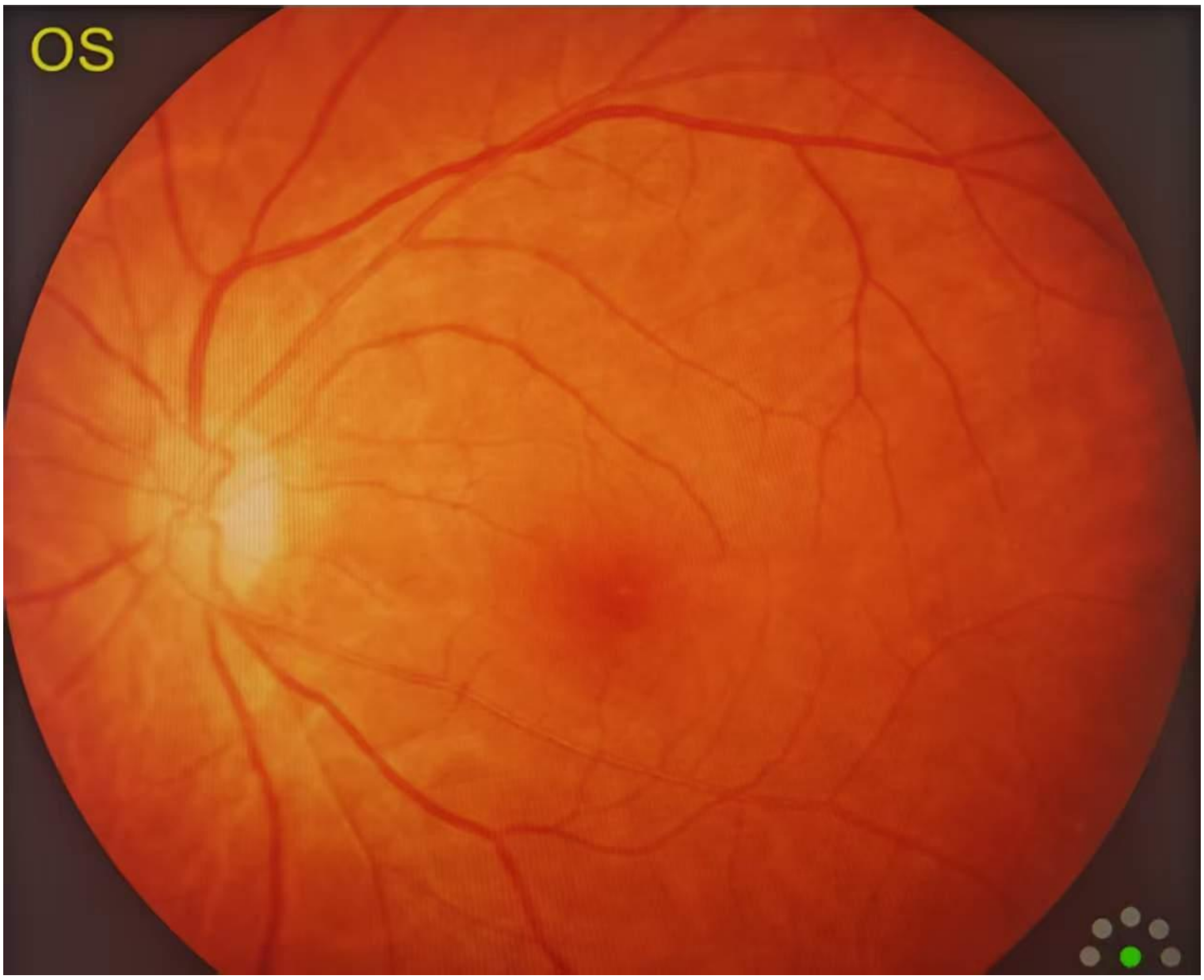

Figure 1 Fundus photographs of the healthy control group

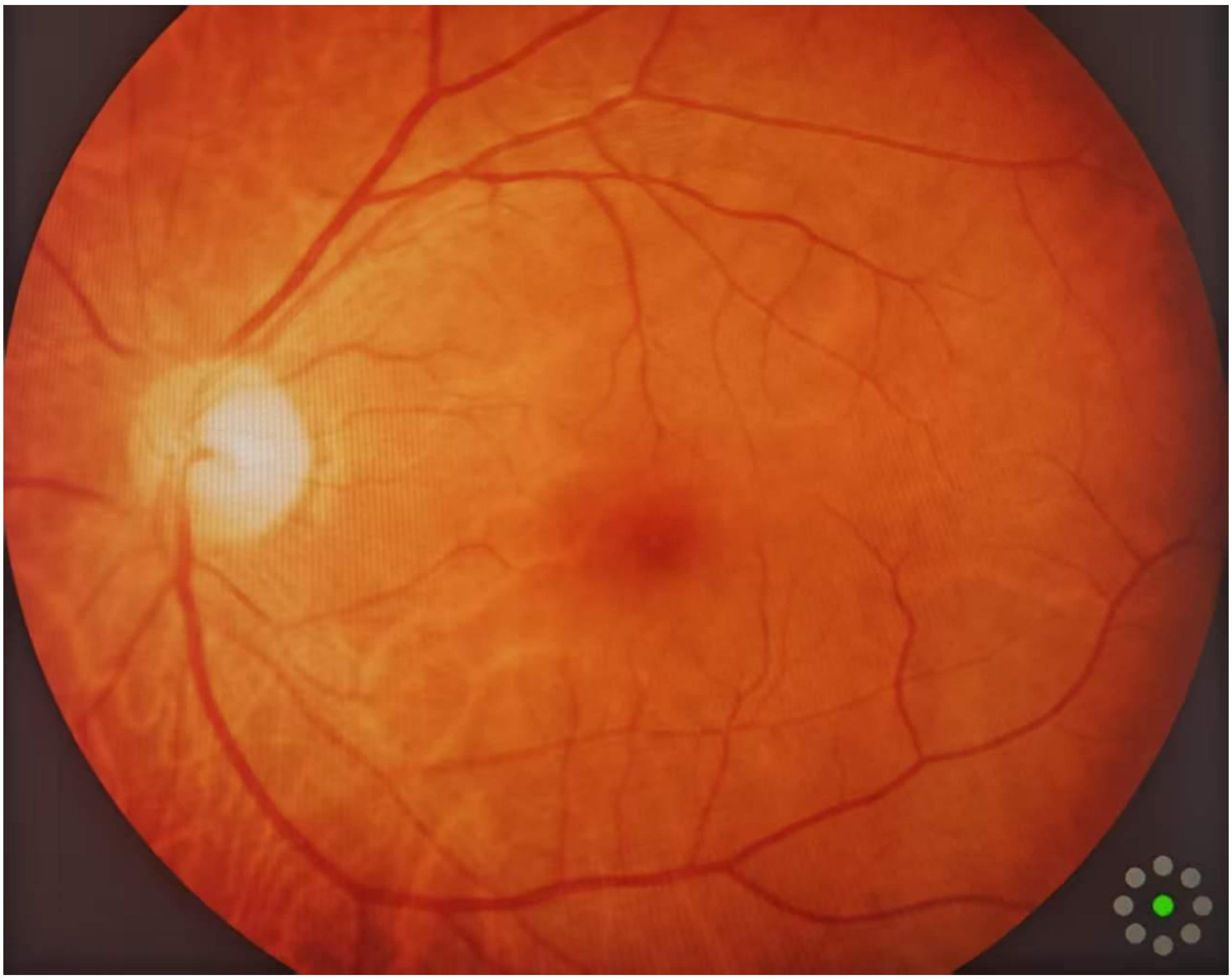

Figure 2 Fundus photographs of the NDR group

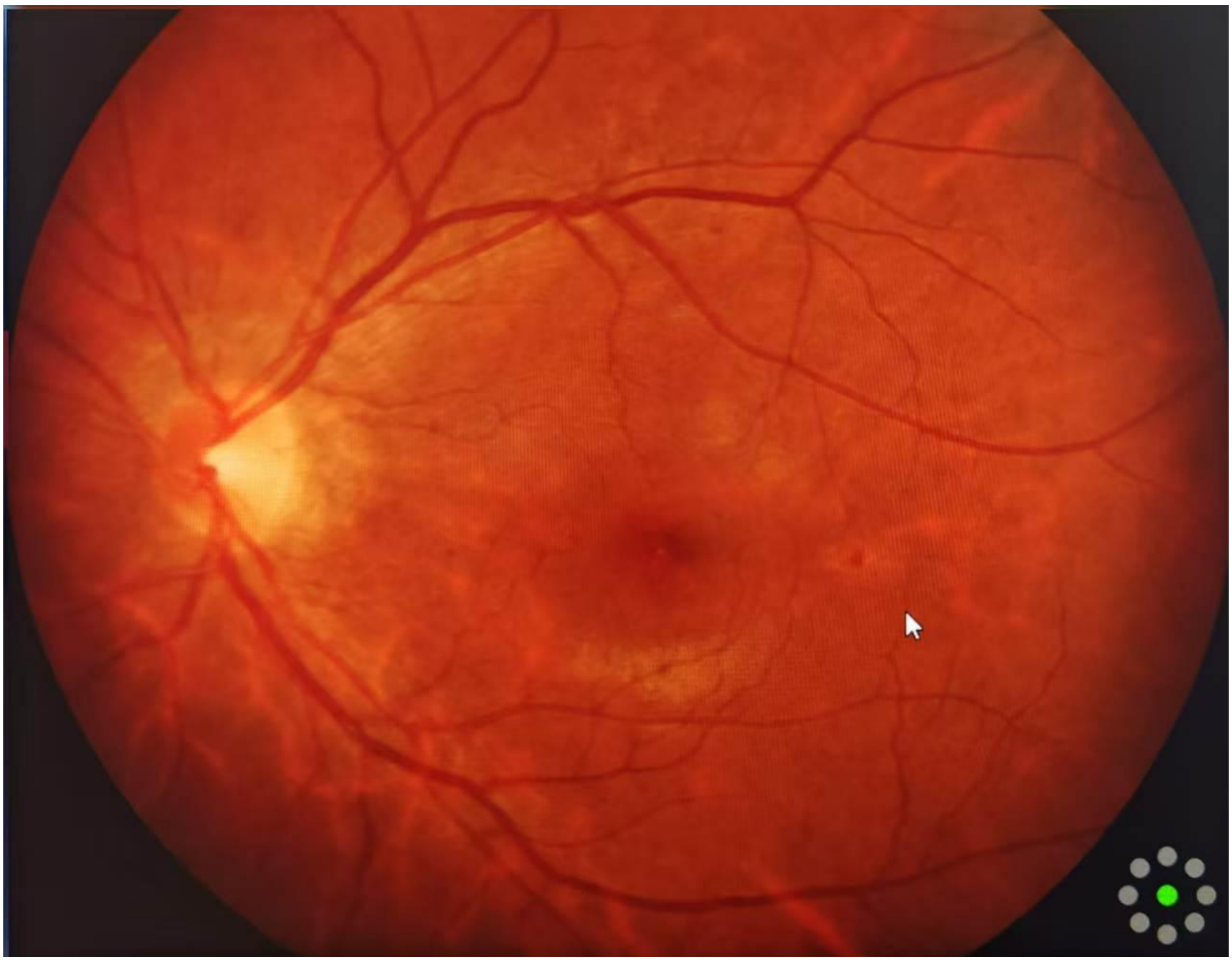

Figure 3 Fundus photographs of the mild NPDR group

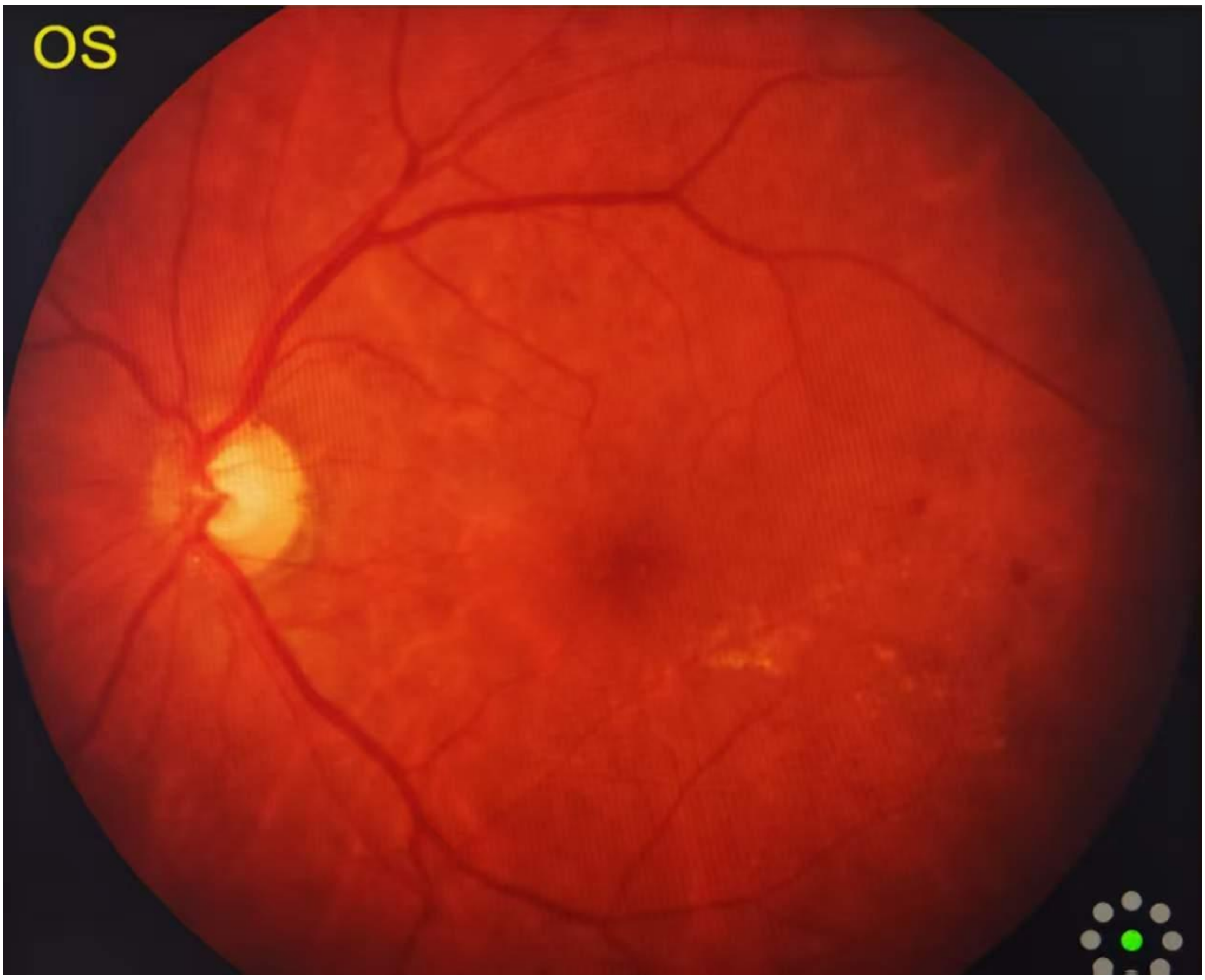

Figure 4 Fundus photographs of the moderate NPDR group

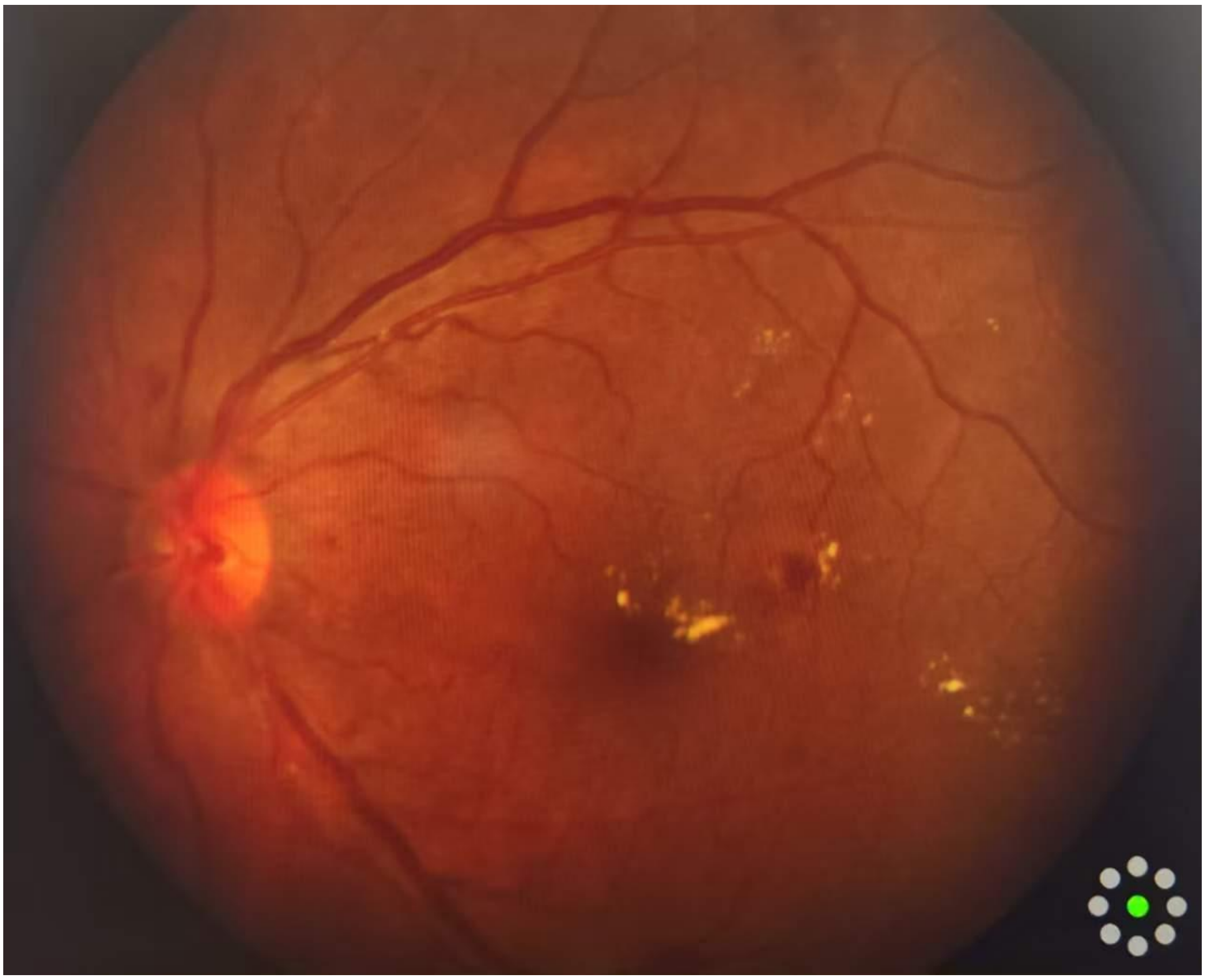

Figure 5 Fundus photographs of the severe NPDR group
