# Supplementary material for: Association between peripapillary OCTA parameters and non-proliferative diabetic retinopathy: a cross-sectional study
Source: Int J Retina Vitreous. 2026 Jul 31;12:142. doi: 10.1186/s40942-026-00914-2 (PMC13715244; doi:10.1186/s40942-026-00914-2)
Supplement: Supplementary file 3 [file 40942_2026_914_MOESM3_ESM.pdf]

Supplementary Table 1: sensitivity analysis

Variable	*Wald* χ^2	*P* Value	*OR*	95%*CI*	
**Model 1**					
NFLVPVD	9.764	0.002	0.859	0.781	0.945
**Model 2**					
SvpVD	5.150	0.023	0.906	0.833	0.987
**Model 3**					
Inferior peripapillary RNFL (3mm zone)(μm)	7.289	0.007	0.975	0.957	0.993
**Model 4**					
Superior peripapillary RNFL (6mm zone)(μm)	6.261	0.012	0.960	0.930	0.991
**Model 5**					
NFLVPVD	5.701	0.017	0.870	0.777	0.975
SvpVD	0.174	0.676	0.978	0.880	1.086
**Model 6**					
Inferior peripapillary RNFL (3mm zone)(μm)	3.114	0.078	0.982	0.962	1.002
Superior peripapillary RNFL (6mm zone)(μm)	1.797	0.180	0.976	0.942	1.011
**Model 7**					
NFLVPVD	5.801	0.016	0.884	0.800	0.977
Inferior peripapillary RNFL (3mm zone)(μm)	3.104	0.078	0.983	0.964	1.002

Sensitivity analysis results

Sensitivity analysis was performed by incorporating all clinical confounding variables (e.g.,age, glycated hemoglobin (HbA1c), duration of diabetes, logMAR visual acuity, body mass index (BMI), total cholesterol (TC), triglycerides (TG), high-density lipoprotein cholesterol (HDL-C), low-density lipoprotein cholesterol (LDL-C), sex, smoking status, and drinking status) into the multivariate model.

Models 1 to 4 performed separate multivariate logistic regression analyses for variables with statistically significant intergroup differences in univariate analysis of peripapillary OCTA parameters. All models were adjusted for all clinically relevant covariates. After adjustment for all variables, the results showed that: peripapillary NFLVPVD (odds ratio [OR] = 0.859, 95% confidence interval [CI]: 0.781–0.945), SVPVD (OR = 0.906, 95% CI: 0.833–0.987), inferior pRNFL thickness at 3 mm (OR = 0.975, 95% CI: 0.957–0.993), and superior pRNFL thickness at

6 mm (OR = 0.960, 95% CI: 0.930–0.991) were each significantly associated with NPDR status. In all cases, lower values of these parameters were observed in the NPDR group.

In Model 5 and Model 6, to mitigate multicollinearity among variables, vascular density indices (peripapillary nerve fiber layer vascular density, NFLVPVD; superficial vascular plexus vascular density, SVPVD) and retinal nerve fiber layer parameters were categorized separately. Regression models adjusted for all clinically relevant confounding covariates were established to compare intergroup differences.Model 7 served as the primary model. Representative parameters of vascular density and retinal nerve fiber layer thickness were integrated to establish a combined multivariate regression model together with all clinically relevant confounding covariates. Although inferior pRNFL thickness at 3 mm showed no statistical significance in Model 6, neurostructural indicators were required for the final analysis; thus, this indicator with a relatively small P value (P=0.078) was selected for inclusion in the multivariate combined model.The findings further verified that NFLVPVD (OR=0.884, 95%CI=0.800–0.977) had an independent association with NPDR status.
